# Supplementary material for: The Use of Machine Learning Algorithms and the Mass Spectrometry Lipidomic Profile of Serum for the Evaluation of Tacrolimus Exposure and Toxicity in Kidney Transplant Recipients
Source: Biomedicines. 2022 May 17;10(5):1157. doi: 10.3390/biomedicines10051157 (PMC9138871; doi:10.3390/biomedicines10051157)
Supplement: Supplementary file 1 [file biomedicines-10-01157-s001.zip › Supplementary Table S1.pdf]

High Tacrolimus Group

	Sex	Age	Tac (ng/mL)	Cr (mg/dL)	Chol (mg/dL)	TG (mg/dL)	ASAT (U/L)	ALAT (U/L)	GGT (U/L)	TB (mg/dl)	Glycemia (mg/dl)	Total proteins(mg/dl)	Ca^{2+} (mmol/L)	Cl^- (mmol/L)	Na^+ (mmol/L)	Potassium (mmol/L)	Amylases (U/L)	Mg^{2+} (mmol/L)	UA (mg/dl)
1	F	44	9,3	1,33	249	188	16,00	18,00	20,00	0,28	95,00	6,70	4,45	110,00	144,00	4,90	121,00	1.52	5,8
2	M	39	9,5	1,94	168	362	31,00	48,00	51,00	0,41	111,00	7,70	4,19	108,00	141,00	4,50	51,00	1.32	7.8
3	M	51	8,3	1,13	145	128	35,00	58,00	105,00	1,15	96,00	6,70	5,16	107,00	144,00	4,30	77,00	1,64	9.5
4	M	63	8,2	2,23	149	90	24,00	27,00	26,00	0,43	150,00	6,80	4,48	113,00	146,00	4,70	80,00	1.53	8.8
5	M	52	8,3	1,69	195	105	20,00	22,00	31,00	0,56	90,00	6,30	4,95	107,00	142,00	4,60	78,00	1,66	5,6
6	M	45	11,9	1,41	211	242	27,00	36,00	39,00	0,66	84,00	6,60	5,30	105,00	142,00	4,10	89,00	1,74	8.4
7	M	46	9,1	0,97	211	64	25,00	26,00	20,00	0,97	91,00	6,80	4,42	105,00	142,00	4,30	95,00	1.53	6,4
8	M	51	8,2	1,41	186	100	11,00	13,00	34,00	0,73	108,00	7,60	4,36	106,00	144,00	4,10	59,00	1.45	5,7
9	M	34	15,9	1,69	119	165	13,00	27,00	15,00	0,67	107,00	7,10	3,99	111,00	140,00	4,00	63,00	1.27	7.8
10	F	41	9,8	1,24	251	139	15,00	13,00	17,00	0,50	94,00	7,20	4,27	106,00	143,00	4,20	95,00	1.58	6.3
11	M	52	8,2	1,23	138	71	16,00	25,00	22,00	0,47	79,00	6,50	4,87	107,00	144,00	5,20	135,00	1,84	5,8
12	M	40	9,7	1,29	234	140	21,00	16,00	23,00	1,86	110,00	7,40	4,52	105,00	144,00	4,10	128,00	1,64	7
13	F	49	9,8	1,46	266	192	21,00	16,00	26,00	1,17	108,00	6,80	4,37	105,00	138,00	3,70	105,00	1.59	6.5
14	M	52	11,6	2,7	483	437	18,00	39,00	49,00	0,52	116,00	7,60	4,45	105,00	139,00	5,00	133,00	1,68	7.5

15	M	59	9,1	2,3	294	189	22,00	30,00	39,00	0,73	110,00	6,60	6,21	100,00	139,00	4,60	110,00	1.34	10.7
16	M	26	8,9	2,56	233	118	32,00	60,00	43,00	0,53	83,00	7,10	4,63	106,00	142,00	4,30	93,00	1,71	8.7
17	M	35	8,4	1,52	271	189	11,00	10,00	61,00	0,80	91,00	6,90	4,24	106,00	143,00	3,90	87,00	1.58	7.4
18	F	57	8,8	0,83	315	97	13,00	6,00	21,00	0,76	112,00	6,70	4,22	107,00	142,00	3,90	97,00	1,81	5
19	F	38	8,2	1,22	214	252	15,00	20,00	13,00	0,46	89,00	7,20	4,23	106,00	136,00	4,60	75,00	1.59	6.9

Abbreviations: Tac – tacrolinemia; Cr – creatinine; Chol – cholesterol; TG – triglycerides; ASAT – aspartate aminotransferase; ALAT – alanine aminotransferase; GGT – gamma-glutamyltransferase; TB – total bilirubin; Na+ – sodium; Cl- – chloride; Ca2+ – ionized calcium; Mg2+ – magnesium; UA – uric acid;

Low Tacrolimus Group

	Sex	Age	Tac (ng/mL)	Cr (mg/dL)	Chol (mg/dL)	TG (mg/dL)	ASAT (U/L)	ALAT (U/L)	GGT (U/L)	TB (mg/dl)	Glycemia (mg/dl)	Total proteins(mg/dl)	Ca2+ (mmol/L)	Cl- (mmol/L)	Na+ (mmol/L)	Potassium (mmol/L)	Amylases (U/L)	Mg2+ (mmol/L)	UA (mg/dl)
1	M	46	4,5	L-TAC	0,94	191	119	14,00	14,00	30,00	1.02	97,00	6,80	4,18	107,00	145,00	3,90	59,00	1,88
2	F	56	3,9	L-TAC	0,84	211	192	19,00	12,00	18,00	0,41	156,00	6,80	4,56	112,00	142,00	4,70	69,00	1.35
3	F	43	2,9	L-TAC	1,39	169	124	15,00	9,00	13,00	1,23	90,00	6,10	4,54	108,00	142,00	4,60	60,00	1,86
4	M	39	4,1	L-TAC	1,2	259	307	23,00	55,00	58,00	0,47	87,00	6,10	5,39	104,00	141,00	3,90	60,00	1,72
5	M	29	4,2	L-TAC	5,23	153	33	10,00	6,00	24,00	0,37	84,00	6,80	3,62	119,00	142,00	6,60	142,00	2,39
6	F	48	4,7	L-TAC	0,86	268	213	39,00	29,00	62,00	0,29	86,00	6,20	5,44	103,00	141,00	4,00	118,00	1.41

7	F	42	4,5	L-TAC	0,89	250	112	20,00	36,00	63,00	0,63	90,00	6,90	4,15	106,00	141,00	4,20	119,00	1,99
8	F	42	4,6	L-TAC	1,05	206	83	17,00	10,00	11,00	0,67	81,00	7,20	4,14	107,00	140,00	4,80	107,00	1,95
9	M	56	4	L-TAC	1,30	159	95	12,00	7,00	20,00	0,49	91,00	7,50	3,77	111,00	142,00	4,40	111,00	1,8
10	F	30	4,3	L-TAC	0,91	200	196	19,00	13,00	11,00	0,44	83,00	6,50	4,48	107,00	142,00	4,30	88,00	1,81
11	M	42	4,5	L-TAC	1,13	289	92	13,00	13,00	15,00	1,34	86,00	6,50	4,39	107,00	140,00	3,90	74,00	1,76
12	M	38	4,7	L-TAC	2,19	223	249	16,00	7,00	29,00	0,72	90,00	6,80	4,32	104,00	138,00	4,20	95,00	1,66
13	M	59	4,6	L-TAC	1,45	134	87	14,00	15,00	11,00	0,88	123,00	7,20	4,32	104,00	141,00	4,10	41,00	1,79
14	F	62	4,9	L-TAC	0,75	200	154	26,00	40,00	41,00	0,94	125,00	6,90	4,29	103,00	142,00	3,70	39,00	1.54
15	M	61	4	L-TAC	2,37	190	153	39,00	39,00	24,00	0,86	428,00	6,90	4,24	102,00	140,00	5,50	46,00	1,98
16	F	56	4,9	L-TAC	2,64	199	135	16,00	20,00	85,00	0,42	131,00	6,40	4,62	107,00	136,00	4,40	95,00	1,68
17	F	50	3,9	L-TAC	1,14	210	124	29,00	53,00	31,00	0,68	98,00	7,60	4,36	99,00	139,00	4,40	107,00	2,06
18	M	54	4,8	L-TAC	1,81	204	278	17,00	12,00	36,00	0.89	101,00	7,50	4,39	108,00	140,00	4,60	115,00	1,93
19	M	45	4,2	L-TAC	1,99	192	76	14,00	11,00	20,00	0,95	108,00	6,60	4,30	108,00	143,00	4,10	136,00	1,61
20	M	38	4,2	L-TAC	2,17	252	179	12,00	6,00	34,00	0,81	200,00	6,40	4,23	105,00	138,00	4,60	66,00	1,69
21	F	51	3,7	L-TAC	0,67	190	64	13,00	6,00	22,00	0,58	93,00	7,10	4,12	106,00	140,00	4,00	57,00	1,62
22	M	48	4,5	L-TAC	2,33	186	138	30,00	21,00	12,00	1,16	91,00	7,30	4,33	105,00	140,00	5,30	78,00	1.5
23	M	41	4,2	L-TAC	1,37	250	187	16,00	19,00	16,00	0,47	75,00	6,60	4,25	107,00	142,00	3,60	68,00	2,06

Abbreviations: Tac – tacrolinemia; Cr – creatinine; Chol – cholesterol; TG – triglycerides; ASAT – aspartate aminotransferase; ALAT – alanine aminotransferase; GGT – gamma-glutamyltransferase; TB – total bilirubin; Na^+ – sodium; Cl^- – chloride; Ca^{2+} – ionized calcium; Mg^{2+} – magnesium; UA – uric acid;
