# Supplementary material for: The Use of Machine Learning Algorithms and the Mass Spectrometry Lipidomic Profile of Serum for the Evaluation of Tacrolimus Exposure and Toxicity in Kidney Transplant Recipients
Source: Biomedicines. 2022 May 17;10(5):1157. doi: 10.3390/biomedicines10051157 (PMC9138871; doi:10.3390/biomedicines10051157)
Supplement: Supplementary file 1 [file biomedicines-10-01157-s001.zip › Supplementary Table S2.pdf]

1. Steroid metabolism

m/z	Metabolite
271,1851	Estrone
301,1397	Andrenosterone
363,3099	Cortisol
365,2763	Tetrahydrocortisone
377,3259	18-Oxocortisol
403,2318	Cortisone acetate/24 hydoxycholesterol
413,2712	25-Hydroxyvitamin D2
465,3781	Testosterone glucuronide
481,2596	11-Oxo-androsterone glucuronide
497,1988	Pregnanediol-3-glucuronide
569,3498	Deoxycholic acid 3-glucuronide
579,2911	1,25-Dihydroxyvitamin D3 3-glycoside
598,4631	14:0 Cholesterol ester
619,4393	16:3 Cholesterol ester
679,509	20:1 Cholesterol ester
702,2074	22:4 Cholesterol ester

2. Aminoacids and derivatives

m/z	Metabolite
149,021	2-Hydroxyglutarate
203,0504	Dimethyl arginine
269,2073	Dl Homocystine
385,2919	S-adenosyl homocysteine
406,328	N-nonadecanoyl taurine
407,3359	linolenoyl-glutamine
410,3469	N-(9,12-octadecadienoyl)-glutamic acid
430,3771	N-Oleoyl phenylalanine
432,2811	N-stearoyl phenylalanine
487,3221	Glucosylgalactosyl hydroxylysine
526,2904	3,5-Diiodothyronine

3. Acylcarnitines

m/z	Metabolite
245,0782	Valerylcarnitine
290,2702	Adipoylcarnitine
370,2938	cis-5-Tetradecenoylcarnitine
384,3454	3-hydroxytetradecadienoylcarnitine
396,3309	hexadecadienoylcarnitine
414,3206	Heptadecanoyl carnitine
424,3628	Linoleoyl carnitine
438,3788	hidroxyoctadecatrienoil carnitine
440,357	hydroxylinoleoylcarnitine
458,3468	O-(17-carboxyheptadecanoyl)carnitine
472,3561	Cervonyl carnitine
476,3932	docosatetraenoylcarnitine
480,3437	docosadienoylcarnitine
484,3819	O-behenoylcarnitine

4. Faty acids and esters

m/z	Metabolite
227,1261	2-tetradecenoic acid C14H26O2
239,2011	Pentadecynoic acid (C15:4)
267,2671	Norlinoleic acid C17H30O2
279,1596	gamma linoleic acid C18H30O2
281,1727	Linoelaidic acid C18H32O2
313,2728	Eicosanoic (arachidic) acid C20H40O2
326,3771	N-oleoyl ethanolamine
331,2831	Docosapentaenoic acid C22H34O2
333,2992	Docosatetraenoic acid C22H36O2
335,2781	Docosatrienoic acid C22H36O2
338,3411	Erucamide (docosenamide) C22H43NO
343,2936	Eicosanedioic acid C20H38O4
349,2936	Tetracosapentaynoic acid C24H28O2
350,3261	eicosatrienoyl-ethanolamine C22H39NO2
352,3048	eicosadienoyl)-ethanolamine C22H41NO2
359,3151	Tetracosapentaenoic acid C24H38O2
391,2832	hexacosatrienoic acid C26H46O3
399,3077	N-Palmitoyltryptamine
419,2954	Palmitoyl glucuronide
501,3254	Palmitoleyl linolenate & isom.C34H60O2
507,328	Oleyl palmitate & isomers C34H66O2
509,3971	Stearyl palmitate
529,3979	Linoleyl stearate
531,3854	Linolenyl stearate
533,2781	Stearyl linoleate/Oleyl oleate C36H68O2
535,1155	Stearyl oleate/Arachidyl palmitoleate C36H70O2

5. Metabolic derivatives of glycerophospholipids

m/z	Metabolite
185,1127	Phosphorylcholine
341,303	9-Hexadecenoylcholine
435,2597	LPA(0:0/18:2)
437,3225	LPA(0:0/18:1)
452,371	LPE 16:1
454,3718	LPE 16:0
459,2913	LPA(20:4/0:0)
466,401	LPC(14:1)
468,3888	LPC(14:0)
482,4048	LPC 15:0
485,3511	LPA(22:5)
487,3598	LPA(22:4)
494,3324	LPC 16:1
496,3916	LPC (16:0)
516,3716	LPC(18:4)
518,3586	LPC(18:3)
520,379	LPC(18:2)
522,3538	LPC(18:1)
523,3046	LPA(24:0)
524,3699	LPC(18:0)
526,4306	LPS(18:0)
528,4111	LPE(22:5)

541,2618	DG(16:0/14:0/0:0)
542,3209	PC(20:5)
544,3481	LPC (20:4)
546,3947	LPC(20:3)
568,3448	LPC(22:6)
570,4558	LPC(22:5)
573,4609	DG(15:1/18:4)
575,4141	DG(15:1/18:3)
586,4491	DG(18:3/16:2)
588,4136	DG18:4/16:1/0:0)
590,4252	DG18:4/16:0/0:0)
591,38	PA(14:1/14:0)
591,38	PA(14:1/14:0)
595,3795	DG(18:1/16:0/0:0)
597,4535	DG(16:0/18:0/0:0)
603,4034	DG(15:0/20:4
606,4545	LPC(24:1)
608,4696	LPC(24:0)
**611,3**	DG(18:4(6Z,9Z,12Z,15Z)/18:3
613,338	DG(18:3/18:3)
627,4708	DG(18:2n6/0:0/18:4n3)
**634,455**	PE(28:1)
635,4127	PA(31:0)
**639,4351**	PA(32:5)
641,4802	DG(18:1/20:5/0:0)
642,5112	DG(20:5/18:1/0:0)
644,4897	DG(20:5/18:0/0:0)
648,4063	DG(20:3/18:0/0:0)
650,4721	DG(18:0/20:2/0:0)
652,4887	DG(18:0/20:1/0:0)
657,3706	PA(18:3/15:0)
**658,5053**	PE(18:3/12:0)
670,5089	DG(18:1/22:5/0:0)
672,4597	DG(18:1/22:4/0:0)
674,4986	DG(18:1/22:3/0:0)
740,5394	PE(18:3/18:1)
758,5623	PS(16:0/18:3)
760,4609	PE(18:3/20:5)
760,5781	PC (16:0/18:1)
762,9459	PC (16:0/18:0)
764,5422	PS(16:0/18:0)
766,5622	Phosphatidyl glycerol PG(18:4/18:3)
778,5318	PC(18:3/18:3)
780,4848	PS(18:4/18:2)
780,5461	PC(18:3/18:2)
782,5218	PS(18:3/18:2)
782,5629	PC(18:2/18:2)
784,531	PS(18:2/18:2)
784,5776	PC(18:1/18:2)
785,4532	PS(16:0/20:4)
806,9759	PC(20:4/18:2)

828,5875	PC(22:5/18:4)
832,5733	PC(22:4/18:3)
834,5921	PC(22:4/18:2)
888,6487	PS (22:4/22:4)

6. Ceramides si sfingomielines

m/z	Metabolite
366,3229	C17 Sphingosine-1-Phospahte
380,3368	C18 Sphingosine 1-phosphate
394,3525	C19 Sphingosine-1-phosphate
510,4274	Cer(d18:1/14:0)
512,4222	Cer(d18:0/14:0)
540,4258	Cer(d18:0/16:0)
556,4393	Cer(t18:0/16:0)
564,3575	Cer(d18:1/18:1)
566,4355	Cer(d18:0/18:1)
584,4453	Cer(t18:0/18:0)
600,4655	Cer(t18:0/18:0(2OH))
614,4807	Cer(t18:0/19:0(2OH))
628,4532	Cer(t18:0/20:0(2OH))
656,4896	Cer(t18:0/22:0(2OH))
677,3897	SM(d18:0/14:0)
679,4429	Cer(d18:1/26:0)
681,2601	Cer(d18:0/26:0)

7. Purines and polar derivates

m/z	Metabolite
476,3044	2',3'-Dideoxyadenosine-5-triphosphate/
502,3726	D-glucosaminide
525,2893	6-Thioinosine triphosphate

8. Proinflamatory lipids

m/z	Metabolite
319,2835	18-HEPE
321,1318	12-HETE
329,1584	Prostaglandin M
**353,2668**	Prostaglandin E2/D2
355,2811	Prostaglandin H1/D1/F2
369,2433	Thromboxane B3

9.Retinoids

m/z	Metabolite
463,3018	Retinyl beta-glucuronide
525,374	Retinyl palmitate
547,3417	all-trans-retinyl linolate
551,3528	all-trans-retinyl oleate

10. Tacrolimus metabolits

m/z	Metabolite
804,5415	Tacrolimus (parent ion)
790,5773	13-O--Demethyl tacrolimus (or 15 izom) parent ion

776,2237	13,15- didemethyl tacrolimus (parent ion)
786,5916	fragment Tacrolimus (product ion)
770,5913	fragment Tacrolimus (product ion)
768,5798	fragment Tacrolimus (product ion)
751,5103	fragment Tacrolimus (product ion)
696,5118	fragment Tacrolimus (product ion)
772,5752	fragment 13-O-Demethyl Tacrolimus (product ion)
756,5470	fragment 13-didemethyl tacrolimus (product ion)
