# Supplementary figures and images for: The Use of Machine Learning Algorithms and the Mass Spectrometry Lipidomic Profile of Serum for the Evaluation of Tacrolimus Exposure and Toxicity in Kidney Transplant Recipients
Source: Biomedicines. 2022 May 17;10(5):1157. doi: 10.3390/biomedicines10051157 (PMC9138871; doi:10.3390/biomedicines10051157)

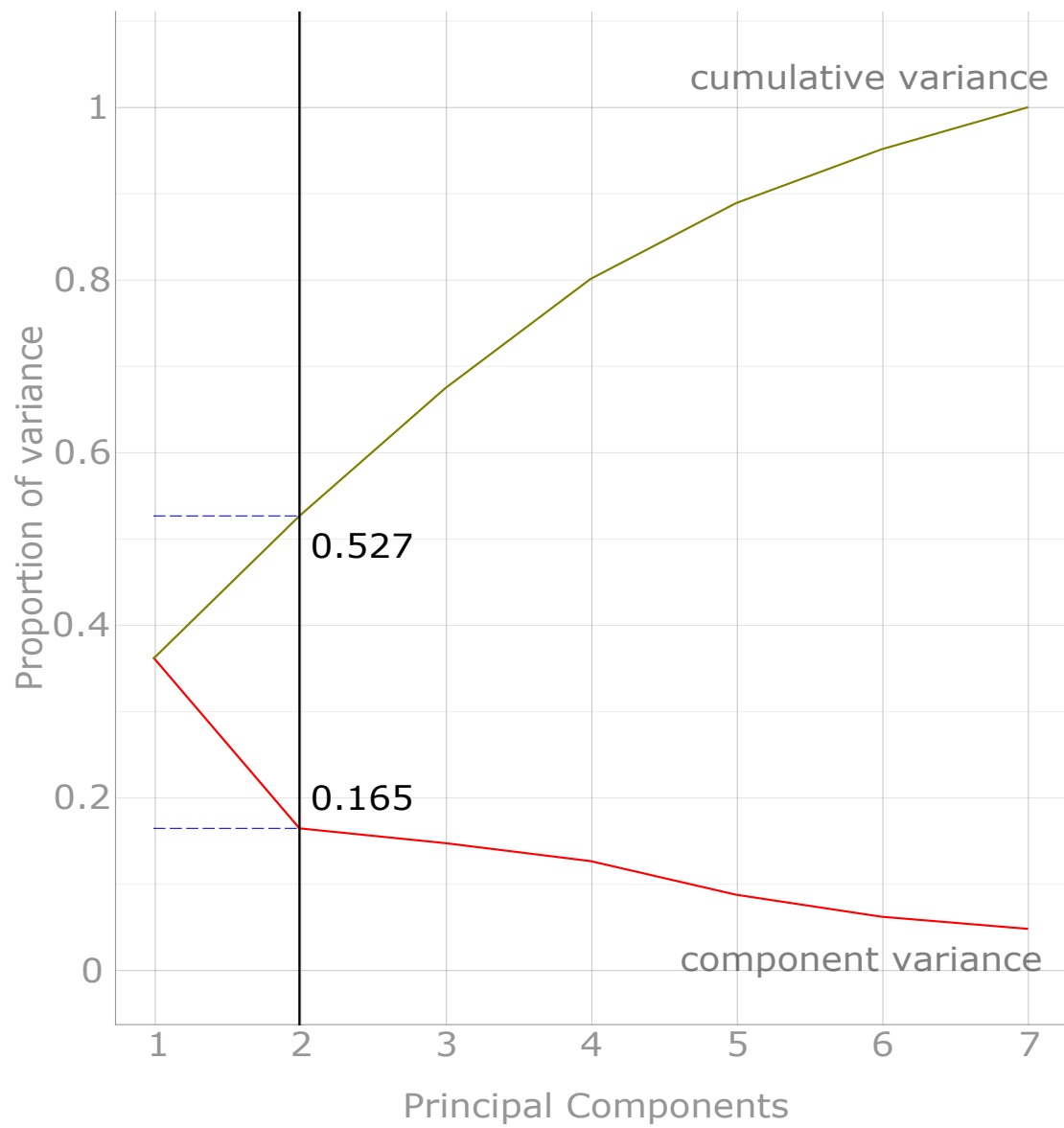

Supplement: Supplementary file 1 [file biomedicines-10-01157-s001.zip › Supplementary Figure S1.pdf]
